# Diabetes prevalence and its risk factors in urban Pondicherry

**DOI:** 10.4103/0973-3930.57348

**Published:** 2009

**Authors:** Sanjay Kumar Gupta, Zile Singh, Anil J. Purty, Mohan Vishwanathan

**Affiliations:** Department of Community Medicine, Pondicherry Institute of Medical Sciences, Kalapet, Pondicherry-605 014, India; 1 Madras Diabetes Research Foundation (MDRF), 6B Conran Smith Road, Gopalapuram, Chennai-600 086, India

**Keywords:** Diabetes mellitus, Indian Diabetes Risk score, obesity, risk factors, urban area

## Abstract

The present study was conducted in the Urban Health centre, Muthialpet, Pondicherry covering a population of 40000 from July to October 2007 by using a predesigned and pretested proforma to find out the risk of diabetes in general population by using Indian Diabetes Risk Score. A total of 616 respondents were studied comprising 325(53%) females and 290(47%) males. Majority 518(85%) were Hindus. Of them, 380 (62 %) had studied up to higher secondary and above, 539 (87%) belonged to upper middle and upper socioeconomic class. A large number of the subjects 422(68%) were above 35 years of age. Most of the respondents 558(90.50%) indulged in mild to moderate physical activity. Again, 422 (68.50%) had no family history of diabetes mellitus, 315 (51%) individuals were in the overweight category (>25 BMI), and 261 (83%) of high Diabetic Risk Score individuals were overweight. It is observed that chances of high diabetic score increase with the increase in BMI. Prevalence of diabetes in the studied population were 51 (8.27%), out of that 39 (76%) had high (>60) IDRS score. The relationship between BMI and IDRS shows that if BMI increases from under weight (<18.50) to obesity (>30) chances of risk for diabetes also increases significantly.

## Introduction

Although great efforts have been made by developed countries to control infectious diseases, noncommunicable diseases have not received the same attention. Diabetes mellitus is one of the noncommunicable diseases which have become a major global health problem. The International Diabetes Federation (IDF) estimated that currently there are 100 million people with diabetes worldwide representing about 6% of all adults.[1] This figure is predicted to reach 240 million by 2010.[1] Asia is one of the regions that have high prevalence of diabetes and it is estimated that 20% of current global diabetic population resides in South-East Asia Region. Indeed, the number of people with diabetes in India is likely to double in less than 2 decades, from 39.9 million (in 2007) to 69.9 million by 2025.[2,3] The population in India has an increased susceptibility to diabetes mellitus. The Indian Council of Medical Research (ICMR) study done in the 1970s reported a prevalence of 2.3% in urban areas[4,5] which has risen to 12 -19 % in 2000s. Correspondingly, in rural areas, prevalence rates have increased from around 1%[4,5] to 4 - 10%, and even 13.2% in one study.[6] Thus, it is clear that both in urban and rural India, prevalence rates of diabetes are rising rapidly with a rough urban-rural ratio of 2:1 or 3:1 being maintained through the last 2 -3 decades with the exception of Kerala where rural prevalence rates have caught up with or even overtaken urban prevalence rates.[7] A balanced approach to improve awareness about diabetes among both the patients and the medical fraternity is critical. Although improving control of diabetes in India is important, the associated risks of tight control in high risk groups should also be kept in mind.[8]

Most prevalence studies in India have come from large metropolitan cities[9] and some from rural areas.[10] Data is needed from smaller towns. Pondicherry is a union territory that is ideally suited for epidemiological studies.

### Aim

The aim of this study is to assess the risk of diabetes mellitus in adults above the age of 20 years in urban Pondicherry using the Indian Diabetes Risk Score (IDRS) developed by Mohan *et al*.[11]

The objectives are as follows:

1. To study the prevalence of diabetes in a urban Pondicherry population
2. To estimate the usefulness of the Indian diabetes risk score for detecting high risk cases for diabetes in urban area of Pondicherry.
3. To compare prevalence of risk factors for diabetes among the known diabetic subjects and those without diabetes.

## Materials and Methods

This is a cross-sectional (descriptive) study carried out in the field practice area of rural health centre (UHC) Muthialpet, department of Community Medicine, Pondicherry Institute of Medical Sciences, Pondicherry, covering the total population of 40 000. From the center, four wards were selected by purposive sampling, and all population above the age of 20 years, presented on the day of survey and willing to participate were taken as a sample population for the study. The total number of selected subjects were 700 (13%- non - responder) and surveyed sample from all four wards was 616. The duration of survey was from July to October 2007. In all subjects, family history of diabetes was obtained and details on physical activities and other parameters were assessed using a validated questionnaire.[12] Waist measurements were obtained using standardized technique. Socio-economic status was assessed according to modified BG Prasad classification based on CPI of April 2006[13] and grade of physical activity assessed by asking the following question (A) How physical demanding is your work (occupation)? (B) Do you exercise regularly in your leisure time? (C) How would you grade your physical activity at home? Than combined score of A+B+C = >3 vigorous/ strenuous, 2 moderate, 1 mild, 0 sedentary was calculated. Analysis for high risk was done as per Indian Diabetes Risk Score (IDRS) developed by Mohan *et al*,[11] and parameters comprising two modifiable (waist circumference, physical activity) and two nonmodifiable risk factors (age, family history) for diabetes.[11] IDRS analysis was done using all the four parameters:

if age <35 years score = 0; if 35-49 years score = 20; if >50 years; score = 30; waist circumference < 80 cm for female and <90 cm for male then score = 0; >80-89 cm for female and >90-99 cm male score = 10; > 90 cm for female and >100 cm for male score = 20; physical activities vigorous exercise or strenuous work score= 0; moderate exercise work /home = 10; mild exercise work/home = 20; no exercise and sedentary work/home = 30; family history of diabetes, no family history = 0, family history present either parent = 10, both parents = 20. After adding all four parameters high risk score (>60 very high risk, 30-50 moderate risk, <30 low risk) was helpful to identify subjects at high risk for diabetes and also raised awareness about diabetes and its risk factors.

No ethical issues were involved as no intervention was carried out; however, verbal consent was obtained to proceed with the survey

## Results

A total of 616 respondents were interviewed. Of these, 325 (53%) were females. Majority 518 (85%) were Hindus and a majority of them 539 (87%) belonged to upper middle and upper social class [Table 1]. Majority of males 167(58.50%) had waist circumference of >90 cm, 296 (90%) females with high risk score had waist circumference >90 cm. According to the physical activity, most of them 558 (90.50%) belong to mild to moderate category. Majority 422 (68.50%) of the respondents had no family history of diabetes mellitus. A total of 310 (50.32%) subjects were at moderate risk (IDRS 30-50) for diabetes and 192(31.20%) had high risk for diabetes (IDRS>60) [Table 1]. Significant difference observed between diagnosed cases of diabetes mellitus had high (>60) IDRS score (76%) than in general population (31.0%). Prevalence of overweight (BMI >25) were 315 (51%). The chances of high diabetes risk score are low 3 (6.38%) among individuals who are underweight (BMI < 18.50) than obese, BMI (>30) 53 (44.54%), difference between two groups are significantly high [Table 2]. The prevalence of diabetes in urban area of Pondicherry was 51 (8.27%), out of these cases 39 (76%) had high IDRS score [Table 3].

**Table 1:** Distribution of respondents according to sociodemographic profile and IDRS score

Category	Number	Percentage
Age group		
20-35 years	194	31.49
36-49 years	211	34.25
>50 years	211	34.25
Sex		
Male	290	47.08
Female	326	52.92
Religion		
Hindu	518	84.9
Muslim	14	2.27
Christian	84	13.6
Educational status		
Illiterate	98	15.9
Primary to middle	138	22
Higher secondary	170	27.6
Graduate	210	34.1
Socioeconomic status		
Upper class	439	71.03
Upper middle	100	16.23
Middle	40	6.50
Lower middle	35	5.68
Lower	2	0.32
IRDS score category		
60 (very high risk)	192	31.20
30-50 (moderate risk)	310	50.32
<30 (low risk)	114	18.48

**Table 2:** Distribution of respondents according to their body mass index and Indian Diabetes Risk score

Body Mass Index	Indian Diabetes Risk Score				
	
	Low	Moderate	Very high	Total	
< 18.50 (Underweight)	18 (38.29)	26 (25.98)	3 (6.38)	47 (100%)	*P*<0.00
18.5-24.99 (Normal range)	66 (25.98)	128 (50.39)	60 (23.62)	254 (100%)	
25-29.99 (Preobese)	24 (12.24)	96 (48.97)	76 (38.80)	196 (100%)	
30 and above (Obese)	6 (5.04)	60 (50.42)	53 (44.54)	119 (100%)	*P*<0.00
Total	114 (18.50)	310 (50.32)	192 (31.18)	616 (100%)	

Risk for diabetes significantly increases with the increase in BMI from normal to obese stage (*P*< 0.05); Figures in parentheses are in percentage

**Table 3:** Distribution of respondents according to their known status of diabetes and IDRS score

Known cases of diabetes (N = 616)		High IDRS score in known diabetic (N = 51)	
	
Number	Percentage	Number	Percentage
51	8.27	39	76.47

Prevalence of diabetes in studied population was 8.27%, out of these cases significant percentage (76.47%) had high IDRS score (>60).

## Discussion

In this study, we used simplified Indian Diabetes Risk Score for identifying newly diagnosed high risk subjects in the urban Pondicherry. This is of great significance as use of such scoring system can prove to be a cost effective tool for the screening of diabetes. Further use of such a risk score would be of great help in developing countries like India where there is a marked explosion of diabetes and over half of them remain undiagnosed. (31.2%) of population had high risk score (>60) for diabetes [Table 1]. In a similar study conducted at Chennai by Mohan *et al*, 43% of population was found in high risk category. This risk difference may be due to variance in life styles of the population as our study was conducted in urban Pondicherry whereas Mohan *et al*, conducted the study in a Metropolitan city. Prevalence for most risk factors was very high among known diabetics compared to people with IDRS >60, it retrospectively proved that if you do not reverse prevalence of risk factors, one is likely to get diabetes.

Further confirmation with GTT is required among subjects with IDRS >60 to detect occurrence of diabetes early. Besides this, lifestyle and dietary modification have to be initiated to reverse the risk factors among these groups.

Various studies in the west used different diabetes risk score, based on simple anthropometric, demographic and behavioral factors to detect undiagnosed diabetes.[14–17] We also used diabetes risk score suitable for detecting undiagnosed diabetes in South Asia. The risk score used in this study are those recommended by American Diabetes Association.[18] Compared to other studies, IDRS score has the following merits: its use is simple, scores are easily obtainable and have been drawn from high-risk population. In addition, the score is developed from representative sample of a large metropolitan city of India, the demographic of which is similar to rest of the India. According to the study “Urban rural differences in prevalence of self-reported diabetes in India” people with sedentary lifestyle had diabetes.[19] In our study, we also found that people with sedentary and mild physical activity had a higher risk for diabetes. According to the study conducted by Ramachandran *et al* in urban area of south India,[20] 47% of the people who had diabetes had a positive family history while in our study only 12% of the respondents gave a positive family history. This difference may be due to different life styles and socioeconomic status of the respondents.

## Conclusion

This study provides a use of simplified Indian Diabetes Risk Score for identifying high risk for diabetic subjects in a community. Simplified diabetes risk score has categorized the risk factors based on their degree of severity. In India, mass screening of high risk cases for diabetes can be made cost effective with regular use of IDRS.
